# The Challenges of COVID-19 for People Living With Diabetes: Considerations for Digital Health

**DOI:** 10.2196/19581

**Published:** 2020-05-15

**Authors:** Anissa Gamble, Quynh Pham, Shivani Goyal, Joseph A Cafazzo

**Affiliations:** 1 Centre for Global eHealth Innovation Techna Institute University Health Network Toronto, ON Canada; 2 Institute of Health Policy, Management and Evaluation Dalla Lana School of Public Health University of Toronto Toronto, ON Canada; 3 Institute of Biomaterials and Biomedical Engineering University of Toronto Toronto, ON Canada

**Keywords:** diabetes, digital health, COVID-19, pandemic

## Abstract

The coronavirus disease (COVID-19) is a global pandemic that significantly impacts people living with diabetes. Diabetes-related factors of glycemic control, medication pharmacodynamics, and insulin access can impact the severity of a COVID-19 infection. In this commentary, we explore how digital health can support the diabetes community through the pandemic. For those living with diabetes, digital health presents the opportunity to access care with greater convenience while not having to expose themselves to infection in an in-person clinic. Digital diabetes apps can increase agency in self-care and produce clinically significant improvement in glycemic control through facilitating the capture of diabetes device data. However, the ability to share these data back to the clinic to inform virtual care and enhance diabetes coaching and guidance remains a challenge. In the end, it requires an unnecessarily high level of technical sophistication on the clinic’s part and on those living with diabetes to routinely use their diabetes device data in clinic visits, virtual or otherwise. As the world comes together to fight the COVID-19 pandemic, close collaboration among the global diabetes community is critical to understand and manage the sustained impact of the pandemic on people living with diabetes.

The coronavirus disease (COVID-19) is a global pandemic and significantly impacts individuals living with diabetes. In China, Wu and McGoogan [1] reported that people living with diabetes who contracted the virus had a more than triple mortality rate of 7% in comparison to 2% in those without diabetes. These figures align with previous global pandemics, which were also associated with increased morbidity and mortality in people with diabetes [2]. During the 2009 H1N1 pandemic, Canadians living with diabetes had triple the risk of hospitalization and quadruple the risk of intensive care unit admissions [3]. The 2003 severe acute respiratory syndrome epidemic also resulted in increased hospitalization and disease severity for people with diabetes [4,5]. As global pandemics continue to occur and the prevalence of diabetes increases [6], the diabetes community will be increasingly confronted with ongoing public health challenges [7].

The World Health Organization has warned that older adults and those with pre-existing medical conditions like diabetes are at higher risk of COVID-19 exposure, complications, and death [8]. Since the majority of the diabetes population are older [9] and have multiple comorbidities of obesity, emphysema, hypertension, and heart failure [10,11], they are at greater risk of viral infection. Although data on COVID-19 presentation has yet to support an increased risk of viral contraction in people living with diabetes [12,13], evidence suggests that they may have worse outcomes should they contract the virus [13,14].

Poor glycemic control is a significant contributor to COVID-19 severity. Hyperglycemic events can lead to diabetes ketoacidosis, which is a life-threatening condition that interferes with the immune response to mitigate sepsis and recovery [15]. Coronaviruses have also been shown to bind to their target cells through angiotensin converting enzyme-2 (ACE2). Fang et al [16] proposed that the expression of ACE2 is substantially increased in people managing their diabetes with ACE inhibitors and antihyperglycemic angiotensin II type-I receptor blockers [17]. As such, these individuals may be at an increased risk of developing severe and fatal COVID-19. To maintain adequate glycemic control, people living with diabetes are normally encouraged to eat well, exercise, and maintain good mental health [18-20]. However, efforts to minimize the risk of exposure to COVID-19 have required social distancing and quarantine practices that may exacerbate insulin sensitivity through lower levels of physical activity, abrupt changes in social routine, poor dietary diversity, and diabetes distress [21-24].

Guidelines authored by prominent diabetes societies encourage the use of insulin as the preferred treatment during the global pandemic [25,26]. However, the impact of COVID-19 on the global economy has compromised insulin production and access [27]. For people who are insulin-dependent, the risk of an insulin shortage or delayed delivery is deadly [28]. Health professionals are recommending people to have a 30-day supply of diabetes medication and supplies for their medical devices [29]. This advice may prove difficult to heed for the growing population of people in both urban (10.8%) and rural (7.2%) settings who experience socioeconomic disparities, specifically lower income, as they may not be able to afford adhering to such guidelines [13,30,31]. In addition, the shortage of commercial antibacterial products may impede sterilization techniques for insulin injections and blood glucose monitoring, and promote infection [32]. Significant decreases in traditional in-person clinic availability will require people to adopt and adjust to receiving digital diabetes care [33].

In response to social distancing guidance, outpatient diabetes clinics and family medicine practices have greatly curtailed their services to only the most urgent cases [34]. Even as restrictions are expected to ease over time, there will be continued caution in visiting clinics. In light of these circumstances, the use of previously restricted forms of communication between providers and their patients have been allowed. Most forms of audio, video, or texting technology have been allowed by jurisdictions through not only relaxing privacy and security requirements but also reimbursing providers for these services. Even telephone calls have become an accepted modality for conducting a clinical visit, allowing those without sophisticated consumer devices like smartphones to access services [35-37].

For those living with diabetes, this is an opportunity to be able to access care with greater convenience while not having to expose themselves to infection in an in-person clinic. If the use of virtual visits continues after the pandemic eases―as they are expected to [38]―it opens up a great opportunity to provide more timely access to not only physician care but services that are often scarce for those living with diabetes [39]. With physical distances no longer a factor, virtualizing the care provided by diabetes educators, dieticians, and specialized mental health professionals could improve access further than what was previously possible with in-person encounters [40]. These successes can only be realized if broader digital health inequities of access and literacy are addressed within the diabetes community [41].

Perhaps more compelling than improving access to health services through virtual care, digital health apps can also create greater agency in self-care. A series of studies in recent years have demonstrated that diabetes smartphone apps with the ability to capture diabetes data and other self-reported factors can produce clinically significant improvement in glycemic control for both those living with type 1 diabetes and type 2 diabetes [40,42,43]. These outcomes were achieved without the benefit of a provider facilitating care through the app. Additional studies have since shown that outcomes can be further enhanced with the addition of virtual care and the active use of diabetes data sharing to enhance diabetes coaching and guidance [44,45].

Despite the positive enablers for remote diabetes care, the ability to share diabetes device data back to the clinic remains a challenge [46]. As it stands, the current landscape of diabetes device data interoperability is a patchwork of proprietary technologies, open source tools, and restrictive electronic health record (EHR) policies. In the end, it requires an unnecessarily high level of technical sophistication on the clinic’s part and on those living with diabetes to routinely use their diabetes device data in clinic visits, virtual or otherwise [47-49]. This technical burden will simply continue to hamper efforts to facilitate comprehensive virtual care. It continues to be a challenge to convince manufacturers of diabetes devices and EHR vendors to create truly interoperable systems to ease the burden on the diabetes communities [40]. It is hoped that the pandemic further reveals the flaws of the industry’s business tactics to maintain exclusivity and their slow response in addressing the needs of the diabetes community.

As the world comes together to fight the COVID-19 pandemic, close collaboration among the global diabetes community is critical to understand and manage the sustained impact of the pandemic on people living with diabetes. Figure 1 presents a summary of the challenges of COVID-19 for people living with diabetes and the opportunities of diabetes digital health to support them in this time of need. Contribution and access to trusted diabetes resources that can communicate actionable insights on the status of COVID-19 are needed to support the community through these challenging times [12,13,50-55].

**Figure 1:**
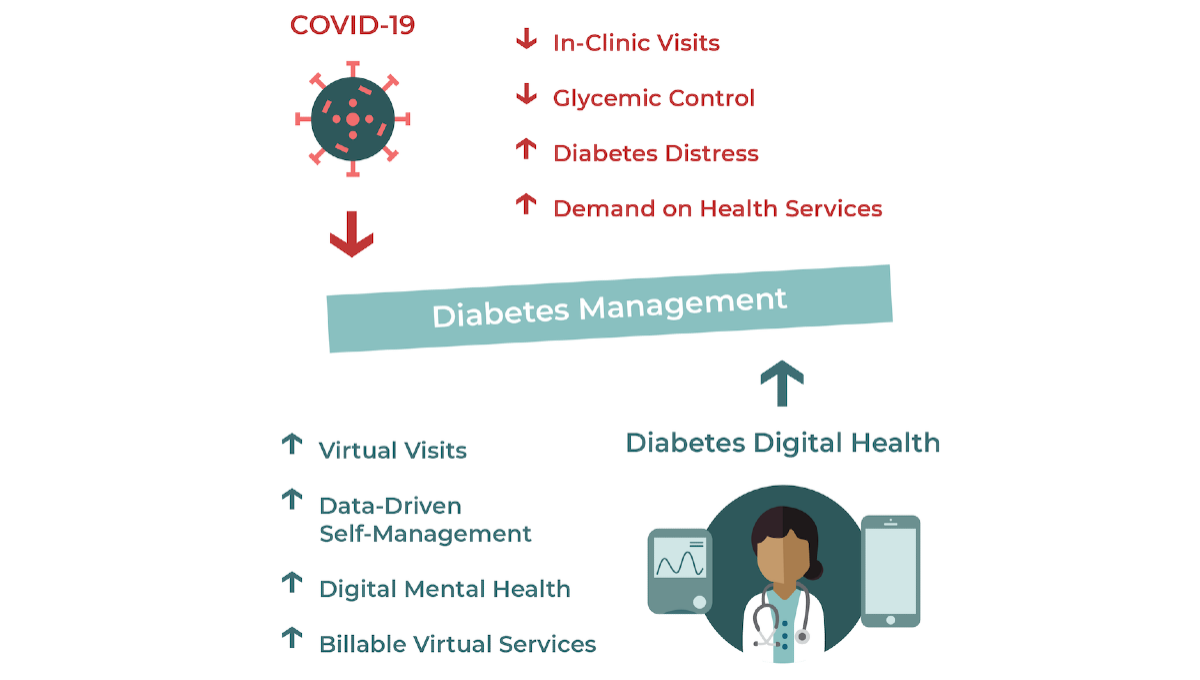
The challenges of COVID-19 for people living with diabetes and the opportunities of diabetes digital health.

## Abbreviations

ACE2: angiotensin converting enzyme-2
COVID-19: coronavirus disease
EHR: electronic health record
